# Associations between sport participation and knee symptoms: a cross-sectional study involving 3053 undergraduate students

**DOI:** 10.1186/s13102-020-00169-w

**Published:** 2020-03-23

**Authors:** Lloyd L. Y. Chan, Arnold Y. L. Wong, Maggie H. Wang

**Affiliations:** 1 School of Public Health and Community Medicine, University of New South Wales, Sydney, NSW 2052 Australia; 2 The Hong Kong Polytechnic University, Hung Hom, Hong Kong SAR, China; 3 JC School of Public Health and Primary Care, The Chinese University of Hong Kong, Shatin, Hong Kong SAR, China; 4 Shenzhen Research Institute, The Chinese University of Hong Kong, Shenzhen, China

**Keywords:** Knee pain, Students, Sports, Youth sports injuries, Athletic injuries, Prevalence

## Abstract

**Background:**

While a number of studies have investigated knee symptoms among elite athletes, few have directly compared the association between engagement in different sports and knee symptoms among young adults in the general population. The current study aimed to investigate the relation between sports participation hours, type/ number of sports engaged, self-rated competitiveness and knee symptoms among undergraduates.

**Methods:**

Undergraduates were invited to participate in a self-administered online survey through invitation emails. Respondents were instructed to provide demographic information (e.g., age, gender, sports participation hours, types of engaged sports, self-rated competitiveness in sports and anxiety level etc.) and to report knee symptoms (current, the last 7 days, the last 12 months, and lifetime). Multiple logistic regressions were conducted to investigate the association between sports participation and current knee symptoms.

**Results:**

Of 17,552 invitees, 3744 responded to the survey. Valid data from 3053 respondents was used for analysis. Forty-four percent of the respondents engaged in sports regularly (≥once per week). Running, cross-training and swimming were the most frequently participated sports among the respondents. The current prevalence rate of knee symptoms was 6.4%. Hours spent participating in combat sports, soccer, yoga, and basketball participation hours were significantly associated with current knee symptoms. Respondents who rated themselves as “competitive” demonstrated a higher risk of having current knee symptoms than “recreational” players. Number of engaged sports was not associated with current knee symptoms among undergraduates.

**Conclusions:**

Certain sports types were associated with current knee symptoms. Compared to self-rated “recreational” players, self-rated “competitive” players were more likely to have current knee symptoms. Students should take preventive measures to minimize their risk of developing knee symptoms, especially when participating in combat sports, soccer, yoga, and basketball, or engaging in sports at a highly competitive level.

## Background

Given the well-known physical and mental health benefits of sports participation, [1, 2] many universities have introduced mandatory sports courses for undergraduate students. However, sports participation is also known to be the major activity leading to injuries that require hospitalization among people aged between 18 and 24 years [3]. A multi-center survey found that 23% of university students in China sustained sports injuries in the past 12 months [4].

Of various collegiate sports injuries, over one-third of them are related to the knee, rendering it to be the most commonly injured body part among undergraduates [5]. Since longer physical activity duration is related to more disabling knee pain among adolescents, [6] it is conceivable that a similar phenomenon may occur in undergraduates. Unfortunately, the relationship between sports participation and knee symptoms among undergraduates remains unclear because prior relevant research mainly focused on children/adolescents [7]. Some common causes of sports-related knee pain in adolescents (e.g., Osgood-Schalatter disease and Sinding-Larsen-Johannson disease) are rare among adults. Hence, findings among adolescents cannot be generalized to young adults.

Knee pain in children and adolescents often persists, [8] and youth knee pain or sports-related knee injuries may increase the risk of future knee osteoarthritis, poor quality of life, and impaired dynamic balance [9, 10]. It is paramount to determine the influences of different sports on knee symptoms in young adults so that appropriate preventive strategies can be implemented.

Recent literature suggests that intensive training in one sport with the exclusion of other sports (sports specialization) may increase the risk of overuse knee injuries, patellofemoral pain, patellar tendinopathy and Osgood-Schlatter disease among high school students [11–13]. However, it remains unclear whether the increased risk is attributed to a single sport participation or an intensive training and high level competition. Further, as previous literature only evaluated the impacts of sports specialization on adolescents, the relation between the number of sports participation or sports competitiveness and knee symptoms among undergraduates remains uncertain.

Additionally, since previous studies only focused on the patterns of knee pain among elite athletes or used a broad term “physical activities” to investigate the relation between various sports activities and musculoskeletal pain, [6, 14] their findings cannot be generalized to a wider population nor be used to determine the effects of different sports activities on knee pain. Without relevant information, it is difficult to develop effective prevention strategies for young non-elite athletes.

Given the above, the present study aimed to: [1] investigate the relation between sports participation hours and current knee symptoms among undergraduates; and [2] determine whether the type and number of sports engaged, and/or self-rated competitiveness of sports engaged were associated with the risks of experiencing knee symptoms among undergraduates.

## Methods

The current cross-sectional survey study was conducted over two periods, September to November 2017, and September to November 2018. Undergraduates of The Hong Kong Polytechnic University and The Chinese University of Hong Kong were invited to participate in an electronic survey through university emails. A follow-up email was sent 2 weeks apart to encourage responses. The introduction page of the survey explained the objectives of the project and the voluntary basis of participation. By submitting the completed electronic survey, respondents gave consent to their participation. This study was approved by the Survey and Behavioral Research Ethics Committee of The Chinese University of Hong Kong (169–17) and the Human Subjects Ethics Subcommittee of The Hong Kong Polytechnic University (HSEARS2017083000), and strictly followed the declaration of Helsinki.

The electronic survey was developed by clinicians and scientists with more than 18 years of experience in clinical practice and/or musculoskeletal research. It comprised two sections. The first section was modified from the Nordic Musculoskeletal Questionnaire, [15] which investigates musculoskeletal symptoms in nine body regions, including neck, shoulder, elbow, wrist/fingers, upper back, lower back, hip/thigh, knee and ankle. Knee symptoms were defined as any acute, sub-acute or chronic symptoms (i.e., ache, pain, discomfort, numbness etc.) that were related to soft tissues (e.g., muscles, tendons, ligaments), joints or bones over the knee region. Participants were instructed to indicate any discomfort at the knee in their lifetime, the past 12 months, the past 7 days, and at the current moment. The second section collected data regarding the participant’s demographics, including gender, age, height, weight, number and type of regular sports participation (i.e., at least weekly in the past 12 months) and average hours of participation in each sport per week in the past 12 months. Participants were asked to indicate their levels of competitiveness in the participated sports as “recreational”, “mainly recreational but occasionally competitive”, “mainly competitive but occasionally recreational” or “competitive”. Taekwondo, Muay Thai, karate, judo and boxing were categorized as “combat sports” for analysis. The questionnaire of the present study is presented in Additional file 1: Appendix 1. Although the maximum number of questions in the survey was 72, only some respondents needed to answer all questions. The average duration for completing the survey was 15 min.

For the statistical analysis, demographic data and other potential risk factors were described in either percentage, or mean and standard deviation (SD). The current, last 7 days, last 12 months, and lifetime prevalence rates of knee symptoms were calculated. The association between the number of participated sports and self-rated competitiveness was analyzed by Chi-Square trend test. Univariate analyses, through simple logistic regressions, were used to compare the characteristics between respondents with and without current knee symptoms. Variables with plausible statistical significance in Wald tests (*p* < 0.05) were included in subsequent multiple logistic regression models. Similarly, current symptoms in other body parts (e.g., neck, lower back or hip regions etc.) and prior history of knee symptoms (knee symptoms occurred more than 12 months ago) between respondents with and without current knee symptoms were compared by univariate analyses. However, these variables were not included in the subsequent multiple logistic regression analyses. It was because these symptoms are expected to be outcomes of other predictors and their adjustments may result in underestimation of effect sizes of other predictors on current knee symptoms [16]. To investigate the association between each specific sports participation and current knee symptoms, a multiple logistic regression analysis was conducted with the presence/absence of current knee symptoms as the outcome and the average hours participating in specific sports per week and variables found significant in the univariate analyses as predictors. Another multiple logistic regression model was conducted with the presence/absence of current knee symptoms as outcome and the number of regularly participated sports, self-perceived competitiveness of each participated sport, variables found significant in univariate analyses and total hours of all types of sports participation as predictors. Statistical analyses were conducted using SPSS version 21 software (IBM, Armonk, NY). A two-tailed significance level of 0.05 was used to denote statistical significance.

## Results

A total of 17,552 invitation emails were sent. The electronic survey was visited by 5666 individuals, of which 3744 respondents completed the survey (overall response rate 21.3%). After the exclusion of duplicates, responses from individuals without valid university email addresses, responses from non-undergraduate students and incomplete responses (Additional file 1: Appendix 2), 3053 cases were included for analysis. The respondents had a mean age of 20.9 years (SD = 2.89 years) and mean BMI of 21.8 (SD = 6.17). Sixty-two percent of the participants were female. Participants’ characteristics were shown in Table 1 and Additional file 1: Appendix 3.

**Table 1:** Characteristics of the respondents included in the study (*N* = 3053)

Variables	
Gender	
Female	1900 (62.36%)
Male	1147 (37.64%)
Age	20.86 (2.89)
Body Mass Index	21.84 (6.17)
Height	165.71 (9.31)
Weight	58.09 (12.96)
Number of regularly participated sports (participate ≥once per week)	
None	1730 (56.77%)
One	894 (29.34%)
Two or more	423 (13.88%)
Average Sports Participation among all respondents, hours per week	2.08 (3.25)
Average Sports participation among respondents who engage in at least one sport regularly, hours per week	4.83 (3.36)
Anxiety, DASS sub-scale	7.94 (3.78)
Depression, DASS sub-scale	6.01 (3.15)
Presence of knee symptoms	
Lifetime	1084 (35.57%)
Past 12 months	708 (23.32%)
Past 7 days	312 (10.24%)
Current	194 (6.37%)
Working surface	
Bed	385 (12.61%)
Desk	2343 (76.72%)
Dining table	325 (10.64%)

Variables are presented in mean (standard deviation) or number of respondents (percentage)

Fifty-seven percent of participants did not engage in sports regularly (i.e. at least weekly in the past 12 months), while 29.3% of them participated in a single sport and 13.9% of them took part in two or more types of sports. The types of engaged sports included badminton, basketball, combat sports (including taekwondo, Muay Thai, karate and judo), cycling, hiking, running, soccer, swimming, table tennis, volley-ball, cross-training, and yoga (Additional file 1: Appendix 4). Running had the highest proportion of participants with more than 10% (*n* = 316) reporting that they engaged in it regularly. Other popular sports included cross-training (6.4%), swimming (6.0%), basketball (5.8%) and badminton (4.9%) (Additional file 1: Appendix 4). Six percent (*n* = 194) of the respondents reported current knee symptoms. The 7-day, 12-month and lifetime prevalence rates of knee symptoms were 10.24, 23.32 and 35.57%, respectively. There was no significant difference in the current prevalence of knee symptoms between females (6.18%) and males (6.72%) (Additional file 1: Appendix 5). Self-rated competitiveness was not significantly associated with the number of regularly participated sports (*p* = 0.30) (Additional file 1: Appendix 6). Respondents with prior history of knee symptoms (7.20%) had significantly higher current prevalence of knee symptoms than those without previous symptoms (3.65%)**.** Participants with current symptoms in neck (OR: 1.46), upper back (OR: 1.80), lower back (OR: 2.85), shoulder (OR: 1.73), elbow (OR: 4.40), wrist/finger (OR: 2.07), hip (OR: 3.49) or ankle (OR: 5.57) regions were at a heightened risk of having current knee symptoms (all *p* < 0.05) **(**Additional file 1: Appendix 5). Respondents who participated in regular sports activities reported a higher current prevalence of knee symptoms (8.6%) than those who did not (4.7%) **(**Additional file 1: Appendix 5).

**Fig. 1:**
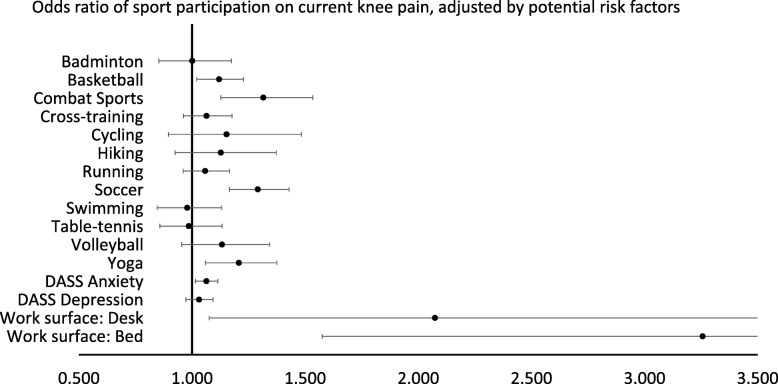
The relations between different sport participation and the current knee symptoms, after adjusting for age, gender and body mass index (*n* = 3053). Odds ratios (95%CIs) were denoted by black dots (horizontal lines). For badminton, basketball, combat sports, cross-training, cycling, hiking, running, soccer, swimming and table tennis, volleyball and yoga, the figure represented adjusted odds ratio of having current knee symptoms for a one hour increase in weekly participation of that sport. For DASS Anxiety sub-scale and Depression sub-scale, the figure represented adjusted odds ratio of having current knee symptoms for one point increase in that sub-score. For work surface: bed and desk, the figure represented the adjusted odds ratio of having current knee symptoms for using bed or desk as work surface, as compared to using dining table. Combat sports, soccer, yoga and basketball, depression level and using desk or bed as work surface were significantly associated with the presence of current knee symptoms

After adjusting for the anxiety level, depression level and the type of work surface, the presence of current knee symptom was significantly associated with the number of hours participating in combat sports (OR: 1.32 per hour, 95%CI: 1.13 to 1.53), soccer (OR: 1.29 per hour, 95%CI: 1.17 to 1.43), yoga (OR: 1.21 per hour, 95%CI: 1.06 to 1.38), and basketball (OR: 1.12 per hour, 95%CI: 1.02 to 1.23) **(**Fig. 1**)**. There was no significant association between the number of regularly participated sports and current knee symptoms (OR: 1.05, 95%CI: 0.67 to 1.67) after adjusting for the anxiety level, depression level, the type of work surface and the self-rated competitiveness in sports **(**Table 2**)**.

**Table 2:** The effects of number of regularly engaged sports, self-perceived competitive levels of sports, and overall duration of sports participation on the current knee symptoms, adjusted by anxiety level, depression level and type of work surface (*N* = 1305)

Variable	Odds Ratio	95% CI of Odds Ratio
Sports participation Time, Hours per Week	1.10*	1.04 to 1.16
Number of sports participated regularly (Reference: One)		
Two or more	1.05	0.67 to 1.67
Self-perceived competitiveness (Reference: Recreational)		
Mainly recreational	1.31	0.80 to 2.14
Mainly competitive	1.19	0.64 to 2.22
Competitive	2.92*	1.67 to 5.10
Anxiety (DASS)	1.07	1.04 to 1.17
Depression (DASS)	1.03	0.96 to 1.11
Work surface (Reference: Dinning Table)		
Desk	3.55	1.14 to 11.1
Bed	2.82	1.00 to 7.92

Significant odds ratio is annotated by asterisk (*)

After accounting for the anxiety level, depression level, the type of work surface, and the number of participated sports, respondents who perceived themselves as “competitive” in sports demonstrated a significantly higher risk of having current knee symptoms (OR: 2.92, 95%CI: 1.67 to 5.10) than those perceived themselves as “recreational” players **(**Table 2**)**.

## Discussion

This is the largest population-based study to evaluate the associations between the type, number, or competitive level of sports activities and current knee symptoms among undergraduate students. Of various reported sports activities, combat sports participation (more hours) was associated with the highest risk of having current knee symptoms, followed by soccer, yoga, and basketball. Our results revealed that undergraduates who self-perceived as “competitive” players had a higher risk of having current knee symptoms than “recreational” players. Furthermore, students participating in a single sport had similar risks of knee symptoms as those participating in multiple sports.

The 12-month and current prevalence rates of knee symptoms among undergraduates in this study were 23.3 and 6.4%, respectively, which concurred with prior research. A recent survey found that the 12-month prevalence of knee symptoms in adults aged between 18 and 40 years was 20.7% [17]. However, since the age range in the prior study was larger than the current study, their results might not truly represent young adults. The current prevalence of anterior knee symptoms among females aged 18–35 years in the USA was reported to be 12% [18]. The higher prevalence of current knee symptoms in their study might be attributed to the gender difference. Specifically, females are at a higher risk of patellofemoral pain syndrome due to differences in movement patterns and anatomical structures [19].

Respondents who participated in regular sports activities reported a higher current prevalence of knee symptoms (8.6%) than those did not (4.7%). This finding was in line with previous studies on younger populations. It was reported that adolescents who participated in a longer duration of leisure sports reported more knee symptoms [20]. Physically active adolescents were also found to have more disabling knee symptoms than physically inactive counterparts [6].

Certain sports were found to increase the risk of knee symptoms. Participation in soccer, basketball, handball and rhythmic and tumbling gymnastics were independent risk factors for overuse knee injuries among children aged between 8 and 15 years [7]. Despite the age differences, some of their findings concurred with ours. Similarly, several injury surveillance studies on US collegiate athletes reported that knee injury rates were the highest among those participating in wrestling, American football, soccer and basketball [21–26]. Both basketball and soccer are organized team sports involving frequent physical contacts and high impact footwork (e.g., sprinting, cutting, pivoting, jumping, and landing), which exert high stress to the knees.

Although the current study indicated a positive association between yoga participation and knee symptoms, inconsistent results have been reported. Incorporating poses, breathing techniques and meditation, yoga practice has been thought to improve muscle strength/flexibility and joint range of motion. Yoga has been widely adopted as one of the effective interventions for improving knee pain, knee joint range of motion, and daily functions among patients with knee osteoarthritis [27]. A large-scale cross-sectional survey involving women aged between 62 and 67 years showed that women who regularly practiced yoga/meditation had a lower prevalence of knee problems [28]. Conversely, Zhu et al. noted that more yoga practice was associated with more meniscus injuries among Chinese women aged between 20 and 49 years [29]. Among yoga-related incidents treated in emergency departments, the median age of patients was 19 years and knee injuries were the most common complaint [30]. These findings, together with the data from the current study, suggest that age may be a moderator between yoga practice and knee injuries. It is hypothesized that younger participants may attempt to try difficult yoga poses that involve more advanced twisting, stretching and near acrobatic components. Therefore, yoga practices may increase the mechanical joint load or cause sprains at the knee, [31] resulting in a higher risk of knee injuries in young adults.

Interestingly, there was no significant difference in the prevalence of knee symptoms between those participating in a single sport and multiple sports. This finding might be ascribed to the fact that most of our respondents were not professional athletes. Therefore, their training intensity and training time (with a mean of 4.83 h per week) might not be high enough to cause overuse syndromes in the knees regardless of the number of involved sports. There was no significant association between the number of regularly participated sports and self-rated competitiveness in sports. The assumption that ‘competitive’ athletes only participate in a single sport may not apply to undergraduates in Hong Kong. Since Hong Kong does not have professional leagues (e.g., National Football League in the USA), many self-rated competitive athletes were only amateur athletes in university sport teams. Therefore, they might not necessarily be trained in a single sport like professional athletes. Some of these ‘competitive’ athletes might participate in multiple sports because of their outstanding motor skills. Future surveys should involve multiple universities in different countries to determine whether our finding can be generalized to undergraduates in other local and/or overseas universities.

The current study recruited a large number of undergraduates with different levels of sports engagements, which enabled the determination of the association between self-perceived competitiveness in sports and knee symptoms. Although competitive players are expected to have better sports skills than recreational players, competitive players in the current study demonstrated a higher risk of experiencing knee symptoms. Since we adjusted for the number of participation hours per week in our analysis, our finding was not explained by a higher exposure to sport. Instead, it might be attributed to the cumulative effects of previous sports-related training/injuries and/or higher training intensity that may outweigh the beneficial effects of better sports skills in preventing knee injuries.

To our knowledge, there was no published report on a head-to-head comparison of the strength of associations between different sport types and knee symptoms in a young adult population. Our participants had a wide spectrum of sports competitiveness, techniques, training volume and intensity. Therefore our findings were more generalizable to young adult populations than past reports on competitive athletes.

Our findings have some important clinical implications. First, undergraduates with current knee symptoms/injuries have an increased risk of having pain in other body regions (e.g., neck, shoulder, elbow, wrist/fingers, upper back, lower back, hip/thigh and ankle). This finding concurred with previous research that workers with knee pain often experienced other concurrent musculoskeletal pain (ranging from 27.1 to 45.0% depending on the body region) [32]. Similarly, high prevalence of co-existing knee and neck symptoms have been reported in a general adult population [33]. While concomitant musculoskeletal complaints and knee pain are common among older adults, [33] our findings suggest that concurrent knee and other joint pain is pervasive among undergraduates. Since altered range of motion or motor control at one joint can adversely affect the function/performance of other joints along the kinetic chain in the upper and lower extremities, [34, 35] the presence of knee pain/injuries can lead to compensatory biomechanics at other joints during sports or daily activities. To prevent or manage concomitant knee and other joint pain, it is necessary to comprehensively detect kinetic chain deficits (e.g., muscle activation sequencing) in upper and lower limbs. Proper kinetic-based regimens (including flexibility, strengthening, endurance, and proprioception training) are needed to restore the optimal muscle activation sequencing, and to prevent excessive loading to particular joints [34]. Second, although participation in certain sports and self-rated competitive athletes may have a higher risk of having current knee symptoms, regular sports participation are important for the physical, mental and social health of young adults [1, 2]. To lower the risk of sports-related knee symptoms/injuries, proper preventive strategies (e.g., proper protective gears for combat sports, screening for history of knee injuries, and/or sports injury prevention education) should be introduced to undergraduates, especially those participating in high-risk sports. Undergraduates should be aware of the potential risk of increased knee symptoms/injuries if they participate in combat sports, soccer, yoga, and basketball. They need to monitor their knee symptoms/injuries and modify their exercise intensity or frequency, if necessary. Third, the higher rate of knee symptoms/injuries among self-rated competitive athletes highlights the importance of allocating more resources to prevent and rehabilitate knee disorders in athletes of high-risk university sports teams.

This study had several limitations. First, the current cross-sectional study could not determine the causal relationship between sports participation and knee symptoms. Second, sports participation volume was summarized as the weekly hours in the past 12 months. It might have been affected by the recall bias and social desirability bias (the tendency of over-reporting sports participation hours as it might be more socially desirable). Third, the influences of sports participation on lifetime and 12-month prevalence of knee symptoms were not investigated because the recall bias might affect the accuracy of these responses. The combined effects may further distort the observed associations. Fourth, there was no official diagnosis of knee problems in the current self-reported study. Although the questionnaire asks a question regarding the diagnosis of their pain, most of the participants did not provide medical diagnosis for their knee symptoms. Without clear diagnoses of knee problems, it may be difficult to understand the underlying mechanisms related to specific sports participation and knee symptoms. Fifth, since the current survey did not inquire the chronicity of knee symptoms, it was impossible to determine which sport participation was more related to acute injuries or chronic knee disorders (e.g., overuse injuries). However, our results have laid the foundation for future prospective cohort study to clarify the casual relation between a particular sport participation and acute or chronic knee symptoms. Future prospective studies are warranted to investigate the underlying mechanisms of some high-risk sports (e.g., combat sports) in causing knee symptoms/diagnoses so that specific prevention strategies can be developed.

## Conclusions

The higher prevalence of knee symptoms among undergraduates engaging in certain sports accentuates the importance of giving sports injury prevention education at the beginning of sports training. Our results have highlighted that combat sports, basketball, soccer, and yoga are significantly associated with current knee symptoms. While the prevalence rates of current knee symptoms did not differ between undergraduates participated in a single sport or multiple sports, a higher risk of current knee symptoms was noted in self-rated competitive athletes.

## Supplementary information

**Additional file 1: Appendix 1** A hardcopy of questionnaire in the current study. **Appendix 2.** Flowchart of participants included in final analyses. **Appendix 3.** Detailed Characteristics of the respondents included in the study. **Appendix 4.** Number of participants and average participation hours in each sport (*n* = 3053). **Appendix 5.** Potential risk factors and association with knee pain analyzed in univariate analysis. **Appendix 6.** Percentage of participants stratified by self-rated competitiveness and number of regularly participated sports (≥1 per week)

## Data Availability

The datasets used and/or analysed during the current study are available from the corresponding author on reasonable request.

## Abbreviations

**BMI**: Body mass index
**CI**: Confidence Interval
**SD**: Standard Deviation
**OR**: Odds ratio
