# Supplementary material for: Associations between sport participation and knee symptoms: a cross-sectional study involving 3053 undergraduate students
Source: BMC Sports Sci Med Rehabil. 2020 Mar 23;12:20. doi: 10.1186/s13102-020-00169-w (PMC7092581; doi:10.1186/s13102-020-00169-w)
Supplement: Supplementary file 1 — Additional file 1: Appendix 1 A hardcopy of questionnaire in the current study. Appendix 2. Flowchart of participants included in final analyses. Appendix 3. Detailed Characteristics of the respondents included in the study. Appendix 4. Number of participants and average participation hours in each sport (n = 3053). Appendix 5. Potential risk factors and association with knee pain analyzed in univariate analysis. Appendix 6. Percentage of participants stratified by self-rated competitiveness and number of regularly participated sports (≥1 per week) [file 13102_2020_169_MOESM1_ESM.docx]

**Appendix 1.** A hardcopy of questionnaire in the current study

You are cordially invited to participate in this online survey conducted by a group of researchers at the Hong Kong Polytechnic University, the Chinese University of Hong Kong and the University of Hong Kong (This research project has been approved by the Survey and Behavioural Research Ethics Committee of The Chinese University of Hong Kong (approval number169-17) and the Human Subject Ethics Subcommittee at The Hong Kong Polytechnic University (approval number: HSEAR20170830003)).

This questionnaire aims to collect data about how often university students experience **musculoskeletal disorders (any acute, sub-acute or chronic symptoms related to muscle, joint, and bone) that were related to soft tissues(e.g. muscles, tendons, ligaments), joint or bone,** and to identify potential **risk factors that may be related to musculoskeletal disorders**. Your participation can help universities prepare better strategies to prevent musculoskeletal disorders in university students.

This questionnaire will **only need 8-15 minutes** to complete. The questions are related to your musculoskeletal disorders at different time periods, causes of your major musculoskeletal disorder, your study and exercise habits, your attitude toward your program, your basic information, any signs of anxiety or depression, and your manual handling knowledge. **Please attempt to complete the survey** and **click on the submit button at the end**. By clicking the submit button, you agree to participate in this survey.

Your answers will be kept strictly **confidential**. You are welcome to give positive and negative feedbacks at the end of this questionnaire. As a token of appreciation, you can enter a lucky draw to win an **iPad mini** or **supermarket coupons**. We are grateful for your participation.

1. In your lifetime, have you experienced any of the following musculoskeletal disorders (i.e., ache, pain, discomfort, numbness) ? *

☐ Head ☐ Neck ☐ Upper back ☐ Lower back ☐ Shoulders ☐ Elbows ☐ Wrists/ Hands ☐ Pelvis/ Groin ☐ Hips/ Thighs ☐ Knees ☐ Lower legs ☐ Ankles/ Feet/ Toes ☐ No (Skip to Q17)

2. In the last 12 months, have you experienced any of the following Musculoskeletal disorders? *

☐ Head ☐ Neck ☐ Upper back ☐ Lower back ☐ Shoulders ☐ Elbows ☐ Wrists/ Hands ☐ Pelvis/ Groin ☐ Hips/ Thighs ☐ Knees ☐ Lower legs ☐ Ankles/ Feet/ Toes ☐ No (Skip to Q17)

3. In the last 7 days, have you experienced any of the following Musculoskeletal disorders? *

☐ Head ☐ Neck ☐ Upper back ☐ Lower back ☐ Shoulders ☐ Elbows ☐ Wrists/ Hands ☐ Pelvis/ Groin ☐ Hips/ Thighs ☐ Knees ☐ Lower legs ☐ Ankles/ Feet/ Toes ☐ No (Skip to Q5)

4. Are you currently experiencing any of the following musculoskeletal disorders? *

☐ Head ☐ Neck ☐ Upper back ☐ Lower back ☐ Shoulders ☐ Elbows ☐ Wrists/ Hands ☐ Pelvis/ Groin ☐ Hips/ Thighs ☐ Knees ☐ Lower legs ☐ Ankles/ Feet/ Toes ☐ No (Skip to Q5)

5. Please indicate the MOST significant musculoskeletal disorder that affect the body region(s) during the last 12 months:

☐ Head ☐ Neck ☐ Upper back ☐ Lower back ☐ Shoulders ☐ Elbows ☐ Wrists/ Hands ☐ Pelvis/ Groin ☐ Hips/ Thighs ☐ Knees ☐ Lower legs ☐ Ankles/ Feet/ Toes

Concerning the musculoskeletal disorder at your [Q5_ANSWER]:

6. What was the cause of your musculoskeletal disorders at [Q5_ANSWER]?

7. What was the average pain intensity of that musculoskeletal disorder at your [Q5_ANSWER]?

1 2 3 4 5 6 7 8 9 10

☐ ☐ ☐ ☐ ☐ ☐ ☐ ☐ ☐ ☐

(With 1 indicating no pain at all, 10 indicating worst possible pain)

8. Have you skipped school because of your Musculoskeletal disorder at [Q5_ANSWER]?

☐Yes ☐No

9. How many days of classes did you skip because of your musculoskeletal disorder at [Q5_ANSWER]

10. Did you seek medical advice for your musculoskeletal disorders in the last 12 months?

☐ Yes ☐ No (Skip to Q12)

11. What kind of medical professionals did you seek?

☐ Other ☐ Osteopath(s) ☐ I prefer not to disclose ☐ General practitioner(s) ☐ Traditional Chinese Medicine Practitioner ☐Orthopaedic ☐ Surgeon(s) ☐ Chiropractors(s) ☐ Physiatrist(s) ☐Physiotherapist(s) ☐Massage therapist(s) ☐Pharmacist(s) ☐Bonesetter(s)

12. How many days did it take for your [Q5_ANSWER] to recover completely?

☐

☐ no recovery at all. I am still having the problem

13. How many times did you experience recurrent musculoskeletal disorder at [Q5_ANSWER] in the last 12 months?

☐

☐ No recurrence

14. Have you considered quitting your current program due to the musculoskeletal disorder at your [Q5_ANSWER]?

☐ Yes ☐No

15. Do you have any prior history of symptoms at [Q5_ANSWER]?

☐Yes ☐No

16. Do you have any family history of musculoskeletal disorder at [Q5_ANSWER]?

☐Yes ☐No

17. Which program are you studying?

☐ Arts ☐ Business ☐Education ☐Engineering ☐Hotel and Tourism Management ☐Medicine ☐Mental Health Nursing ☐Nursing ☐Occupational therapy ☐Optometry ☐Physiotherapy ☐Master in Physiotherapy ☐Radiography ☐Science ☐Social Work ☐Surveying Other

18. In the last academic year, what year of study were you in?

Please fill in 0, if you are a freshman

19. On average in the last academic year, how many hours of lecture per week did you have?

20. On average over the last academic year, how many hours did you engage in discipline-specific practical training per week? (Applicable to healthcare students only)

21. On average over the last academic year, how many hours did you study every week?

22. In the last academic year, how many weeks of full-time clinical placement did you participate? (Applicable to healthcare students only)

Please enter 0 if you didn't have clinical placement.

23. Do you like your current study?

1 2 3 4 5 6 7 8 9 10

☐ ☐ ☐ ☐ ☐ ☐ ☐ ☐ ☐ ☐

1 star indicates you dislike it very much

5 stars indicate that you are Ok with it

10 stars indicates that you like it very much

24. On average over the past 12 months, how many hours per day did you use your smartphone for internet browsing?

25. What kind of computer do you most commonly use? *

☐Other ☐Laptop ☐Tablet ☐Desktop

26. On average over the past 12 months, how many hours do you spend on [Q25_Answer] a day?

27. Aside from Laptop, is there any kind of computer you use frequently?

☐No (Skip to Q29) ☐Laptop ☐Desktop ☐Tablet ☐Other

28. On average over the past 12 months, how many hours do you spend on [Q27_Answer] a day?

29. What kind of school bag do you use the most?

☐Backpack ☐ Duffel bag ☐Messenger bag ☐Hand bag ☐Other

30. What kind of work surface do you use the most?

☐ Dinning Table ☐ Desk ☐ Bed ☐Other

31. In the last academic year, how many hours of part-time job per week did you do?

32. In the past 12 months, do you engage in sports activities regularly (i.e. at least weekly)?

☐ Yes ☐ No (Skip to Q40)

33. What type of sports do you engage the most? *

34. In the past 12 months, how many hours do you participate [Q33_Answer] every week?

35. Do you consider your participation in [Q33_Answer] as social or competitive?

☐ Mainly competitive but occasionally social

☐Mainly social but occasionally competitive

☐Social ☐Competitive

36. Are there any other sports you participate regularly in the past 12 months (i.e. at least weekly)?

☐Yes ☐ No (Skip to Q40)

37. Please name those sports

38. In the past 12 months, how many hours do you participate [Q37_Answer] every week?

39. Do you consider your participation in [Q37_Answer] as social or competitive?

☐ Mainly social but occasionally competitive

☐ Mainly competitive but occasionally social

☐Competitive ☐Social

The next 14 questions will help us know how you are feeling. Please select the answer that best describes how you have been feeling during the PAST WEEK. There are no right or wrong answers. Do not spend too much time on any statement

40. I was aware of dryness of my mouth

☐ Did not apply to me at all

☐Applied to me to some degree, or some of the time

☐Applied to me to a considerable degree, or a good part of time

☐Applied to me very much, or most of the time

41. I couldn't seem to experience any positive feeling at all

☐ Did not apply to me at all

☐Applied to me to some degree, or some of the time

☐Applied to me to a considerable degree, or a good part of time

☐Applied to me very much, or most of the time

42. I experienced breathing difficulty (eg, excessively rapid breathing,

breathlessness in the absence of physical exertion)

☐ Did not apply to me at all

☐Applied to me to some degree, or some of the time

☐Applied to me to a considerable degree, or a good part of time

☐Applied to me very much, or most of the time

43. I found it difficult to work up the initiative to do things

☐ Did not apply to me at all

☐Applied to me to some degree, or some of the time

☐Applied to me to a considerable degree, or a good part of time

☐Applied to me very much, or most of the time

44. I experienced trembling (eg, in the hands)

☐ Did not apply to me at all

☐Applied to me to some degree, or some of the time

☐Applied to me to a considerable degree, or a good part of time

☐Applied to me very much, or most of the time

45. I was worried about situations in which I might panic and make a fool of myself

☐ Did not apply to me at all

☐Applied to me to some degree, or some of the time

☐Applied to me to a considerable degree, or a good part of time

☐Applied to me very much, or most of the time

46. I felt that I had nothing to look forward to

☐ Did not apply to me at all

☐Applied to me to some degree, or some of the time

☐Applied to me to a considerable degree, or a good part of time

☐Applied to me very much, or most of the time

47. I felt down-hearted and blue

☐ Did not apply to me at all

☐Applied to me to some degree, or some of the time

☐Applied to me to a considerable degree, or a good part of time

☐Applied to me very much, or most of the time

48. I felt I was close to panic

☐ Did not apply to me at all

☐Applied to me to some degree, or some of the time

☐Applied to me to a considerable degree, or a good part of time

☐Applied to me very much, or most of the time

49. I was unable to become enthusiastic about anything

☐ Did not apply to me at all

☐Applied to me to some degree, or some of the time

☐Applied to me to a considerable degree, or a good part of time

☐Applied to me very much, or most of the time

50. I felt I wasn't worth much as a person

☐ Did not apply to me at all

☐Applied to me to some degree, or some of the time

☐Applied to me to a considerable degree, or a good part of time

☐Applied to me very much, or most of the time

51. I was aware of the action of my heart in the absence of physical exertion (eg, sense of heart rate increase, heart missing a beat)

☐ Did not apply to me at all

☐Applied to me to some degree, or some of the time

☐Applied to me to a considerable degree, or a good part of time

☐Applied to me very much, or most of the time

52. I felt scared without any good reason

☐ Did not apply to me at all

☐Applied to me to some degree, or some of the time

☐Applied to me to a considerable degree, or a good part of time

☐Applied to me very much, or most of the time

53. I felt that life was meaningless

☐ Did not apply to me at all

☐Applied to me to some degree, or some of the time

☐Applied to me to a considerable degree, or a good part of time

☐Applied to me very much, or most of the time

54. Do you feel pressure from your family?

1 2 3 4 5 6 7 8 9 10

☐ ☐ ☐ ☐ ☐ ☐ ☐ ☐ ☐ ☐

No pressure Extreme pressure

55. Do you feel pressure from your peers?

1 2 3 4 5 6 7 8 9 10

☐ ☐ ☐ ☐ ☐ ☐ ☐ ☐ ☐ ☐

No pressure Extreme pressure

56. Do you feel pressure from your study?

1 2 3 4 5 6 7 8 9 10

☐ ☐ ☐ ☐ ☐ ☐ ☐ ☐ ☐ ☐

No pressure Extreme pressure

57. In the past seven days, what was your average night's sleep?

In hours

58. Do you have difficulty falling asleep?

☐ None ☐ Mild ☐Moderate ☐Severe ☐Very Severe

58. What is your gender?

☐ Female ☐ Male ☐ I prefer not to disclose ☐ Other

59. What is your age?

60. What is your height (in centimetres)?

61. What is your weight (in kilograms)

62. Do you currently smoke cigarettes?

☐Yes

☐ Yes, only at rare occasions (Please skip to Q65)

☐ No, but I have smoked before

☐ No, I do not smoke (Please skip to Q65)

63. How many pieces of cigarette do/did you smoke every day?

64. How many years have/had you been smoking?

65. Do you drink alcohol?

☐ Yes

☐ Yes, occasionally only (Please skip to Q67

☐ No, but I were a drinker before

☐ No, I do not drink alcohol (Please skip to Q67)

66. How many units of alcohol do/did you drink per week?

One unit of alcohol is equivalent to

½ pint of ordinary strength beer

½ pint of cider

1 small glass of wine

1 single measure of spirits

1 single measure of aperitifs

67. Do you drink coffee?

☐ Yes

☐ Yes, occasionally only (Please skip to Q69)

☐ No, but I were a drinker before

☐ No, I do not drink alcohol (Please skip to Q69)

68. How many cups of coffee do you drink every day?

69.In terms of academic results, you consider yourself:

1 2 3 4 5 6 7 8 9 10

☐ ☐ ☐ ☐ ☐ ☐ ☐ ☐ ☐ ☐

Low Achiever Moderate Achiever High Achiever

70. Please fill in your University student email address (optional)

71. Would you like us to contact you for future research opportunity?

☐Yes ☐No

72. Do you have anything else to tell us?

☐No ☐Other

**Appendix 2.** Flowchart of participants included in final analyses **
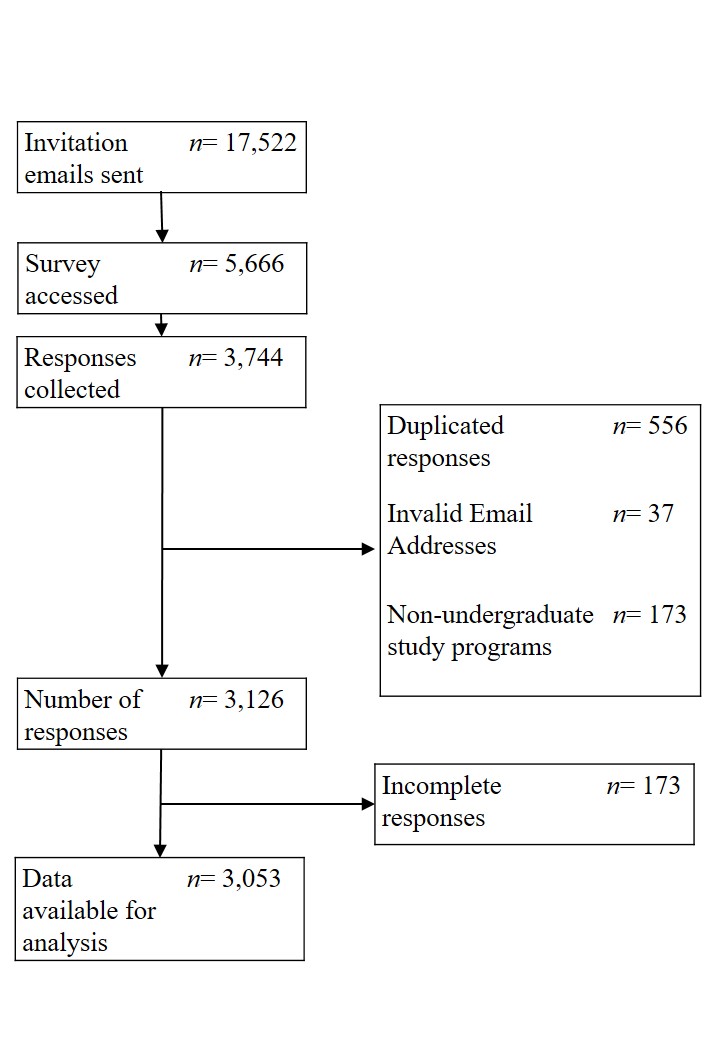
**

**Appendix 3.** Detailed Characteristics of the respondents included in the study (N=3,053)

| Variables | |
| --- | --- |
| Gender | |
| Female | 1900 (62.36%) |
| Male | 1147 (37.64%) |
| Age | 20.86 (2.89) |
| Body Mass Index | 21.84 (6.17) |
| Height | 165.71 (9.31) |
| Weight | 58.09 (12.96) |
| Number of regularly participate sports | |
| None | 1730 (56.77%) |
| One | 894 (29.34%) |
| Two or more | 423 (13.88%) |
| Sports Participation Hours per Week | 2.08 (3.25) |
| Alcohol, unit per week | 0.13 (1.01) |
| Coffee, cup per week | 0.21 (0.52) |
| Tobacco, pieces per week | 0.06 (0.79) |
| Anxiety, DASS sub-scale | 7.94 (3.78) |
| Depression, DASS sub-scale | 6.01 (3.15) |
| Computer usage, hours per day | 4.17 (2.89) |
| Smartphone usage, hours per day | 4.82 (3.54) |
| Self-study duration, hours per day | 18.45 (18.67) |
| Lecture duration, hours per day | 13.89 (9.4) |
| Part-time job, hours per week | 6.85 (11.24) |
| Presence of knee pain |  |
| Lifetime | 1084 (35.57%) |
| Past twelve months | 708 (23.32%) |
| Past seven days | 312 (10.24%) |
| Current | 194 (6.37%) |
| Family pressure, in a scale of 0 to 10 | 4.97 (2.47) |
| Peer pressure, in a scale of 0 to 10 | 5.49 (2.42) |
| Study pressure, in a scale of 0 to 10 | 7.33 (2.21) |
| Opinion of study program , in a scale of 0 to 10 | 6.15 (1.61) |
| Sleeping duration, hours per day | 6.72 (1.35) |
| Year of study | 1.64 (1.47) |
| Living environment |  |
| I prefer not to disclose | 369 (12.09%) |
| Private rental housing | 383 (12.55%) |
| Public rental housing | 837 (27.42%) |
| Residential hall | 566 (18.54%) |
| Self-own apartment | 611 (20.01%) |
| Subsidised home ownership housing | 287 (9.4%) |
| Insomnia |  |
| None | 1272 (41.66%) |
| Mild | 532 (17.43%) |
| Moderate | 939 (30.76%) |
| Severe | 219 (7.17%) |
| Very Severe | 91 (2.98%) |
| Study Program |  |
| Arts | 129 (4.22%) |
| Business | 335 (1%) |
| Education | 5 (0.16%) |
| Engineering | 359 (11.76%) |
| Hotel and Tourism Management | 58 (1.90%) |
| Mental Health Nursing | 65 (2.13%) |
| Nursing | 242 (7.92%) |
| Occupational therapy | 63 (2.06%) |
| Optometry | 16 (0.52%) |
| Physiotherapy | 138 (4.52%) |
| Radiography | 115 (3.77%) |
| Science | 176 (5.76%) |
| Social Work | 19 (0.62%) |
| Surveying | 125 (4.09%) |
| Freshmen | 1208 (39.55%) |
| Working surface |  |
| Bed | 385 (12.61%) |
| Desk | 2343 (76.72%) |
| Dinnig table | 325 (10.64%) |
| Computer type |  |
| Desktop | 556 (18.21%) |
| Laptop | 2218 (72.63%) |
| Tablet | 257 (8.42%) |

Variables are presented in mean (standard deviation)

**Appendix 4.** Number of participants and average participation hours in each sport (n=3,053)

|  | Running | Cross-training | Swimming | Basketball | Badminton | Soccer | Yoga | Volleyball | Combat Sports | Hiking | Table tennis | Cycling |
| --- | --- | --- | --- | --- | --- | --- | --- | --- | --- | --- | --- | --- |
| Number of participants  (percentage of all respondents) | 316  (10.35%) | 196  (6.42%) | 183  (6.00%) | 177  (5.80%) | 144  (4.88%) | 87  (2.85%) | 55  (1.80%) | 55  (1.80%) | 45  (1.51%) | 34  (1.11%) | 34  (1.11%) | 24  (0.79%) |
| Average participation hours  (standard deviation) | 3.61(2.21) | 4.68(2.28) | 3.89(2.32) | 4.32(2.61) | 3.21(2.13) | 4.24(2.80) | 4.15(3.01) | 4.44(2.32) | 3.83(2.45) | 4.38(2.45) | 4.32(2.70) | 3.77(2.30) |

“Taekwondo”, “Muay Thai”, “Karate”, “Judo” and “boxing” were categorized as combat sports. “High intensity interval training”, “gym” and “resistance” were classified into cross-training. “Jogging” and “treadmill” were categorized into running.

**Appendix 5.** Potential risk factors and association with knee pain analyzed in univariate analysis

|  | Without knee pain | With knee pain | OR | 95%CI | p-value |
| --- | --- | --- | --- | --- | --- |
| Age, year | 20.81±2.83 | 20.94±3.24 | 1.01 | (0.97-1.07) | 0.56 |
| Alcohol intake, unit | 0.13±1.02 | 0.13±0.69 | 1 | (0.88-1.15) | 0.96 |
| Smartphone usage, hours per day | 4.82±3.55 | 4.87±3.45 | 1 | (0.96-1.04) | 0.87 |
| Clinical placement, weeks per year | 3.86±6.61 | 3.68±6.37 | 1 | (0.96-1.03) | 0.80 |
| Coffee intake, cup per day | 0.21±0.52 | 0.26±0.6 | 1.18 | (0.94-1.49) | 0.16 |
| Computer usage, hours per day | 4.18±2.89 | 3.97±2.77 | 0.97 | (0.95-1) | 0.32 |
| Family pressure, in a scale of 1 to 10 | 4.97±2.46 | 5.29±2.5 | 1.05 | (0.99-1.12) | 0.08 |
| Anxiety level, DASS anxiety sub-score | 7.87±3.77 | 8.88±3.92 | 1.07 | (1.03-1.11) | <0.01 |
| Depression level, DASS depression sub-score | 5.97±3.14 | 6.57±3.1 | 1.06 | (1.01-1.11) | <0.01 |
| Height, cm | 165.67±9.37 | 166.32±8.27 | 1.01 | (0.99-1.02) | 0.34 |
| Lecture hours, hours per week | 13.91±9.46 | 13.64±8.42 | 1 | (0.98-1.01) | 0.69 |
| Part-time hours, hours per week | 6.77±11.26 | 7.63±10.75 | 1.01 | (0.99-1.02) | 0.31 |
| Peer pressure, in a scale of 1 to 10 | 5.46±2.41 | 5.73±2.4 | 1.05 | (0.99-1.12) | 0.13 |
| Practical training, hours per week | 7.38±15.05 | 9.94±22.07 | 1.01 | (1-1.02) | 0.12 |
| Sleep, hours per night | 6.73±1.34 | 6.53±1.44 | 0.9 | (0.8-1) | 0.05 |
| Tobacco usage, pieces per day | 0.06±0.8 | 0.08±0.65 | 1.03 | (0.88-1.2) | 0.72 |
| Self-study, hours per week | 18.52±18.83 | 16.56±16.28 | 0.99 | (0.99-1) | 0.16 |
| Study pressure, in a scale of 1 to 10 | 7.34±2.2 | 7.51±2.14 | 1.04 | (0.97-1.12) | 0.28 |
| Weight, kg | 58.08±13.14 | 58.37±9.93 | 1 | (0.99-1.01) | 0.76 |
| Year of study | 1.65±1.47 | 1.61±1.45 | 0.98 | (0.89-1.09) | 0.75 |
| Male gender (reference) | 93.38% | 6.72% |  |  |  |
| Female gender | 93.82% | 6.18% | 1.09 | (0.81-1.47) | 0.55 |
| Regular sports engagement  Without (reference) | 95.29% | 4.71% |  |  |  |
| With | 91.39% | 8.61% | 1.90 | (1.42-2.55) | <0.01 |
| Prior history of knee symptoms  Without (reference) | 96.35% | 3.65% |  |  |  |
| With | 92.80% | 7.20% | 2.05 | (1.45–2.89) | <0.01 |
| Current neck symptoms  Without (reference) | 94.06% | 5.94% |  |  |  |
| With | 91.56% | 8.44% | 1.46 | (1.03-2.06) | 0.03 |
| Current upper back symptoms  Without (reference) | 93.98% | 6.02% |  |  |  |
| With | 89.66% | 10.34% | 1.80 | (1.18-2.76) | 0.01 |
| Current lower back symptoms  Without (reference) | 94.56% | 5.44% |  |  |  |
| With | 85.93% | 14.07% | 2.85 | (2.00-4.04) | <0.01 |
| Current shoulder symptoms  Without (reference) | 94.30% | 5.70% |  |  |  |
| With | 90.55% | 9.45% | 1.73 | (1.24-2.40) | <0.01 |
| Current elbow symptoms  Without (reference) | 93.78% | 6.22% |  |  |  |
| With | 77.42% | 22.58% | 4.40 | (1.87-10.34) | <0.01 |
| Current wrist/finger  symptoms  Without (reference) | 93.84% | 6.16% |  |  |  |
| With | 88.03% | 11.97% | 2.07 | (1.16-3.69) | 0.01 |
| Current hip symptoms  Without (reference) | 94.01% | 5.99% |  |  |  |
| With | 81.82% | 18.18% | 3.49 | (2.05-5.94) | <0.01 |
| Current ankle symptoms  Without(reference) | 94.58% | 5.42% |  |  |  |
| With | 75.80% | 24.20% | 5.57 | (3.74-8.30) | <0.01 |
| Work surface  Dining table (reference) | 96.92% | 3.08% |  |  |  |
| Bed | 90.65% | 9.35% | 3.25 | (1.59-6.66) | <0.01 |
| Desk | 93.64% | 6.36% | 2.14 | (1.12-4.1) | 0.02 |
| Type of computer used  Tablet (reference) | 93.39% | 6.61% |  |  |  |
| Desktop | 95.50% | 4.50% | 0.66 | (0.35-1.25) | 0.21 |
| Laptop | 93.15% | 6.85% | 1.04 | (0.62-1.74) | 0.89 |
| Study program  Freshman (reference) | 93.05% | 6.95% |  |  |  |
| Arts | 93.02% | 6.98% | 1 | (0.49-2.05) | 0.99 |
| Business | 94.63% | 5.37% | 0.76 | (0.45-1.28) | 0.3 |
| Education | 100.00% | 0.00% | 0 | (0.00-infinity) | 1 |
| Engineering | 93.59% | 6.41% | 0.92 | (0.57-1.48) | 0.72 |
| Hotel and Tourism Management | 100.00% | 0.00% | 0 | (0.00-infinity) | 1 |
| Mental health nursing | 93.85% | 6.15% | 0.88 | (0.31-2.47) | 0.8 |
| Nursing | 93.80% | 6.20% | 0.88 | (0.5-1.56) | 0.67 |
| Occupational therapy | 92.06% | 7.94% | 1.15 | (0.45-2.95) | 0.77 |
| Optometry | 100.00% | 0.00% | 0 | (0.00-infinity) | 1 |
| Physiotherapy | 94.20% | 5.80% | 0.82 | (0.39-1.74) | 0.61 |
| Radiography | 93.91% | 6.09% | 0.87 | (0.39-1.92) | 0.73 |
| Science | 94.89% | 5.11% | 0.72 | (0.36-1.46) | 0.36 |
| Social work | 94.74% | 5.26% | 0.74 | (0.1-5.64) | 0.77 |
| Surveying | 90.40% | 9.60% | 1.42 | (0.75-2.68) | 0.28 |
| Living environment  Subsidised home ownership housing (reference) | 93.38% | 6.62% |  |  |  |
| I prefer not to disclose | 92.95% | 7.05% | 1.07 | (0.58-1.97) | 0.83 |
| Private rental housing | 94.26% | 5.74% | 0.86 | (0.46-1.62) | 0.64 |
| Public rental housing | 93.19% | 6.81% | 1.03 | (0.6-1.76) | 0.91 |
| Residential hall | 94.17% | 5.83% | 0.87 | (0.49-1.56) | 0.65 |
| Self-owned department | 93.78% | 6.22% | 0.94 | (0.53-1.65) | 0.82 |
| Insomnia  Very severe(Reference) | 91.21% | 8.79% |  |  |  |
| None | 93.55% | 6.45% | 0.71 | (0.33-1.53) | 0.39 |
| Mild | 92.48% | 7.52% | 0.84 | (0.38-1.87) | 0.67 |
| Moderate | 94.36% | 5.64% | 0.62 | (0.29-1.35) | 0.23 |
| Severe | 94.52% | 5.48% | 0.60 | (0.24-1.52) | 0.28 |

**Appendix 6.** Percentage of participants stratified by self-rated competitiveness and number of regularly participated sports (≥1 per week)

|  | Number of regularly participated sports | |
| --- | --- | --- |
|  | **One** | **Two or more** |
| Competitive | 10.43% | 9.03% |
| Mainly competitive but occasionally social | 12.11% | 15.68% |
| Mainly social but occasionally competitive | 26.68% | 30.88% |
| Social | 50.78% | 44.41% |
| Chi-Square trend test, p-value = 0.30 | | |
